# In memoriam Dr. med. Udo Schagen (born September 27, 1939, in Düsseldorf; died June 4, 2026, in Oderaue)

**DOI:** 10.3205/zma001867

**Published:** 2026-09-15

**Authors:** Claudia Kiessling, Kai Schnabel, Andreas Winkelmann

**Affiliations:** 1 Witten/Herdecke University, Chair for the Education of Personal and Interpersonal Competencies in Health Care, Witten, Germany; 2 University of Bern, Institute for Medical Education, Department for Education and Media, Bern, Switzerland; 3 Brandenburg Medical School Theodor Fontane, Institute of Anatomy, Neuruppin, Germany

## Obituary

“It suddenly occurred to me that this would be a unique opportunity to create an entirely new kind of medical school – a medical school that would train not the doctor of the past, but the doctor of the future – the social doctor” (Henry E. Siegrist, 1940) [1], quoted from [2].

This quote opened a tribute by Schleiermacher & Winau in the “Zeitschrift für Medizinische Ausbildung” in 1999 on the occasion of Udo Schagen’s 60^th^ birthday [2]. Today, we bid farewell to our highly esteemed colleague, an important guide and pioneer of reform in medical education. Throughout his professional career, Udo Schagen (Fig. 1) was consistently committed to improving the study of medicine. He was a long-standing member of the DACH Association for Medical Education (GMA), a regular contributor to the “Zeitschrift für medizinische Ausbildung” – the predecessor of GMS J Med Educ [3], [4], [5] – and was one of the founding patrons of the Berlin reformed medical curriculum, the first model program at a public university in Germany [6], [7].

### Professional career

#### Social medicine, health policy, and critical medicine

Udo Schagen studied medicine in Munich, Münster, Innsbruck, and Hamburg, and subsequently worked as a researcher at the Institute of Physiology at Freie Universität Berlin (FU Berlin), where he also earned his doctorate. He developed an interest in social medicine and issues related to medical education at an early stage. Together with other critical doctors, he observed that the psychosocial dimension of health and illness was largely absent from medical education in the 1970s [8], [9], [10]. It remained a constant concern of his to expand medical education to include topics such as social inequality, poverty as a cause of illness, the healthcare system, and social medicine [2]. These demands, which were always grounded in historical context, also contributed to a broader conception of health and illness in line with a biopsychosocial model and to the reorientation of a patriarchal “doctor-patient relationship” toward an appreciation of patients’ lived experiences and psychosocial embeddedness, as well as an encounter on equal terms.

Udo Schagen’s professional career was closely linked to the city of Berlin and its academic and health policy institutions. In 1970, he co-founded a Central Institute for Social Medicine and served first as a planning assistant and then as head of the Medical Planning Group within the Planning Staff and, subsequently, the Department of Medicine under the President of the Free University of Berlin [2]. Starting in 1986, he headed the Center for Contemporary History at the Institute for the History of Medicine at the Free University of Berlin, where his work and research focused on recent medical history, education and continuing education in the health professions, as well as the politics, structure, and development of the healthcare system and medical schools within Germany’s political systems [11]. He initially co-directed the center with Dr. Eberhard Göbel and later with Prof. Dr. Sabine Schleiermacher [https://medizingeschichte.charite.de/], [8]. He was co-editor of the series “Kritische Medizin im Argument,” a member of various working groups and expert commissions, and active at the professional policy level as chair of the Joint Continuing Education Committee of the Berlin Medical Association (including as a long-standing member of the “FrAktion Gesundheit”) as well as on the “Medical Continuing Education” Committee of the German Medical Association [2], [12].

#### Reformed medical education

For us, the authors CK and KS, Udo Schagen also became an important mentor on a personal level. The student initiative “Inhalts AG,” which emerged from a nationwide student strike in 1988/89 aimed at improving medical education [13], [14], sought an institutional affiliation and a place to further develop ideas for a reformed medical curriculum after the strike ended. The Inhalts AG found this home at the Center for Contemporary History, where it was provided with a desk, a computer, and printing facilities, enabling it to carry out the project tutorial – which Udo Schagen supported with great dedication – aimed at implementing the “Berlin Model” [15]. This is all the more commendable given that, in an otherwise very conservative faculty, taking students’ ideas and suggestions seriously – let alone promoting them – was by no means a given. Together with Eberhard Göbel, Udo Schagen organized a seminar on “The Image of the Physician of the Future” (“Arztbild der Zukunft”) to bring the motivated but still relatively inexperienced students up to speed on the reform efforts of the Murrhardter Kreis of the Robert Bosch Foundation [16]. This led to contact with Prof. Thure von Uexküll and his reform ideas regarding integrated biopsychosocial medicine and a (new) theory of human medicine [17], [18]. These concepts became the fundamental pillars of the reformed medical curriculum in Berlin.

Udo Schagen was a dedicated, gifted, and authentic advocate for a patient-centered approach to medicine, both in undergraduate education and in continuing medical education. He possessed the foresight, courage, and tenacity to speak out and voice even uncomfortable truths when necessary. His appearance at the hearing before the Committee on Science and Research in the Berlin House of Representatives on June 13, 1990, was particularly memorable; there, he spoke out clearly against the vice president and the stance of Free University, which was hesitant to support a curriculum reform.

Udo Schagen maintained a friendly connection with the reformed medical program at Charité–University Medicine Berlin – which was finally established in 1999 – as a lecturer and advisor. Here, too, he was particularly committed to exploring with students the principles of medical theory and practice, and especially the psychosocial implications related to health and illness.

#### Medicine and contemporary history

Another major focus of Udo Schagen’s research was the role of medicine under National Socialism [19] and in the postwar period [20]. Together with Sabine Schleiermacher and others, he examined the history of the Charité during the “Third Reich” [21]. Famous figures who worked at Berlin University repeatedly played an important role in this context, like Ferdinand Sauerbruch [22] and Hermann Stieve. Udo Schagen was the first to seriously investigate the Nazi past of Berlin anatomist Hermann Stieve [23], before the discipline of anatomy itself looked into this. Without making much fuss about it, however, he soon engaged one anatomist (the author A.W.) to join in investigating this past [24]. This did not only lead to an amicable working relationship, but also had a sustained influence on further research in this field. Finally, Udo Schagen was also deeply committed to commemorating and honoring marginalized and displaced Charité employees [25].

### In gratitude

Udo Schagen passed away on June 4, 2026. We have lost a wise, dedicated, kind-hearted, and sometimes critical colleague, friend, mentor, and expert – simply a wonderful person. Our deepest sympathies go out to his family and to all who knew him and will miss him. We will miss you!

## Authors’ ORCIDs

- Claudia Kiessling: [0000-0003-4104-4854]
- Kai Schnabel: [0000-0002-6977-2717]
- Andreas Winkelmann: [0000-0003-1047-2477]

## Competing interests

The authors declare that they have no competing interests. 

## Figures and Tables

**Figure 1:**
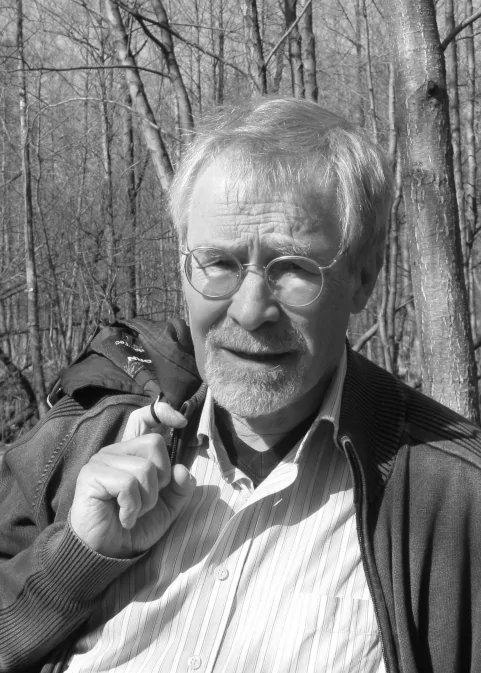
Personal photo of C. Kiessling, 2011
